# Emerging Pollutants in Chinstrap Penguins and Krill from Deception Island (South Shetland Islands, Antarctica)

**DOI:** 10.3390/toxics13070549

**Published:** 2025-06-29

**Authors:** Miguel Motas, Silvia Jerez-Rodríguez, José Manuel Veiga-del-Baño, Juan José Ramos, José Oliva, Miguel Ángel Cámara, Pedro Andreo-Martínez, Simonetta Corsolini

**Affiliations:** 1 Department of Toxicology, Faculty of Veterinary, Regional Campus of International Excellence “Campus Mare Nostrum”, University of Murcia, Campus of Espinardo, 30100 Murcia, Spain; motas@um.es (M.M.); silviajerez@um.es (S.J.-R.); 2 Department of Agricultural Chemistry, Faculty of Chemistry, Regional Campus of International Excellence “Campus Mare Nostrum”, University of Murcia, Campus of Espinardo, 30100 Murcia, Spain; chemavb@um.es (J.M.V.-d.-B.); josoliva@um.es (J.O.); mcamara@um.es (M.Á.C.); 3 National Centre for Environmental Health (CNSA), Instituto de Salud Carlos III (ISCIII), 28029 Madrid, Spain; jjramos@isciii.es; 4 Department of Physical, Earth and Environmental Sciences, University of Siena, Via Mattioli, 4, 53100 Siena, Italy; simonetta.corsolini@unisi.it

**Keywords:** Antarctica, BPA, DEHP, krill, MEHP, PFOA, PFOS, *Pygoscelis antarctica*, South Shetland Islands

## Abstract

This study aimed to evaluate the presence of emerging pollutants [perfluorinated compounds, phthalates and bisphenol A (BPA)] in chinstrap penguins (*Pygoscelis antarctica*) and krill (*Euphausia superba*) from Deception Island (South Shetland Islands, Antarctica) to provide data on the occurrence of emerging pollutants in Antarctica. For this purpose, thirty-four samples were studied, including four samples of adult tissue and six samples of chick tissue, as well as krill samples from the area. The selected samples were subjected to extraction processes and subsequent analytical determination of perfluorooctane sulfonate, perfluorooctanoic acid, di(2-ethylhexyl) phthalate, mono(2-ethylhexyl) phthalate and BPA using high-performance liquid chromatography coupled with electrospray ionization mass spectrometry. Our results highlight that the analyzed organic pollutants, except for BPA, are clearly present in *Pygoscelis antarctica* and *Euphausia superba* from Deception Island.

## 1. Introduction

The Antarctic has long been considered one of the last unspoilt areas on Earth and is used as a worldwide emblem of preservation. This perception has been influenced by its isolation, which is due to natural barriers such as water masses and atmospheric currents. Nonetheless, the notion that this region is wholly unspoilt started to be contested in the 1960s. Sladen et al. [1] were pioneers in the field of environmental science and were among the first to identify the pesticide dichlorodiphenyltrichloroethane and some of its related compounds in tissue samples of Adelie penguins and a crabeater seal collected in Antarctica in 1964. This was remarkable given that pesticides had never been employed on the continent. Subsequent to that, a large number of additional persistent organic pollutants (POPs) have been identified in Antarctic specimens, suggesting that even the most remote region of the globe is not impervious to pollution and its worldwide ramifications [2,3,4]. Also, POPs might get to these places through long-distance air and sea travel, but there seems to be a lot less POPs that get there. In very small amounts, this can also be passed on to animals that live in the sea and birds that migrate [5].

The process by which POPs and other pollutants accumulate in polar regions and remote areas is known as global distillation. This process is driven by two mechanisms: cold condensation and global fractionation [6]. Because of this, scientists from all over the world have become very interested in researching the large amount of pollutants that are found in the environment in Antarctica and how they behave [7].

POPs are a major worldwide contamination problem because they can be transported over long distances, mainly via air currents. These contaminants can travel to the polar regions, where low temperatures and extended periods of darkness allow them to degrade very slowly, resulting in their accumulation in ice [8]. When the ice thaws, these contaminants are released back into the environment, where they have the potential to enter food chains and accumulate in the tissues of organisms, a process known as biomagnification [9]. In addition, global warming and increasing temperatures in specific regions, such as the Antarctic Peninsula, amplify the transportation and deposition of these contaminants.

The Stockholm Convention’s list of POPs is dynamic and includes new compounds, such as per- and polyfluoroalkyl substances (PFASs), which are synthetic chemicals extensively used in many consumer and industrial products due to their multiple applications. PFASs are a family consisting of more than 4000 compounds that have been used since the 1940s. Perfluorinated compounds (PFCs) are made up of a linear or branched chain of carbon atoms of variable length (from four to fourteen). C-F bonds make these molecules particularly resistant to hydrolysis, photolysis or microbial degradation processes. They also possess particular thermal resistance and an exceptional hydro- and lipophobic nature [10,11]. For all these reasons, they are highly persistent in the environment, with a high tendency for bioaccumulation [12]. They have the ability to be transported over long distances, forming a longer, more volatile chain that, once these remote areas are reached, degrades into more stable compounds such as perfluorooctane sulfonate (PFOS) and perfluorooctanoic acid (PFOA) [13]. It is known that these contaminants are present in high concentrations in snow and rain at levels of ng·L^−1^ [14,15].

Phthalates are a family of chemical compounds used in the plastic materials industry as plasticizing agents or as additives to polymers to improve their flexibility and malleability. Polyvinyl chloride (PVC) is the main plastic material in which phthalates are used. With the passage of time and with use, they are released into the environment, which is why phthalates are considered ubiquitous environmental pollutants. Among them, di(2-ethylhexyl) phthalate (DEHP) is the most abundant compound in the environment and its primary metabolite is mono(2-ethylhexyl) phthalate (MEHP) [16].

The presence of phthalates has been documented in remote areas such as the Antarctic marine environment [17,18,19], indicating that long-range transport may be a significant factor in the movement of these compounds from industrial regions to more isolated areas.

Bisphenol A (BPA) is not considered a POP because it does not meet the criteria of persistence, bioaccumulation and toxicity that define POPs. It is an organic compound with two phenol functional groups that is mainly used in association with other substances to produce plastics and resins. This compound and its derivatives have been on the market for more than 60 years. It is used in the synthesis of polyesters, polysulfonates and polyether-ketones, as an antioxidant in some plasticizers and as an inhibitor of PVC polymerization. It is also used as a precursor to flame retardants and as a fungicide. Plasticizers such as BPA have been demonstrated to interfere with the endocrine systems of biota and impede biological processes at negligible concentrations [20]. The presence of bisphenol A has been identified in food packaging and polyethylene terephthalate bottles. It is hypothesized that the substance may be migrating from the plastic products in question and being ingested. The BPA is present in wastewater, derived from human urine and feces, without undergoing any metabolic processes. The presence of BPA has been documented at McMurdo Station, Scott Base and Esperanza Base. In the 2009/10 sampling season, McMurdo and Scott Base reported similar concentrations. However, in the 2012 sampling season, Scott Base reported concentrations that were 30 times higher [21]. BPA concentrations in the treated effluents are much higher elsewhere in the world. In fact, they are about 15 times higher [22].

Sea birds are important for environmental biomonitoring because they are easy to identify, are found everywhere and are part of the ecosystems that live in the sea. They exhibit heightened sensitivity to alterations engendered by human activity within their habitats. Through meticulous scrutiny of their dietary intake, researchers are able to ascertain fluctuations in the prevalence of their prey. These changes offer a glimpse into a variety of concerns, such as shifts in environmental conditions, the influence of human activity on specific species and fluctuations in nutrient consumption, which can serve as a key route for pollutants to enter their systems [23,24].

It has been proposed that Antarctic penguins act as potential eco-sentinels to monitor worldwide ecological contamination [25,26]. As top predators and long-lived species, they are subject to biomagnification and bioaccumulation, which are processes that increase the levels of pollutants in an organism’s body over time. Moreover, they boast extensive distribution ranges, with substantial populations, a sizable body size that makes sampling straightforward and the capacity to combine contamination over both time and space [27]. Also, the different species of penguin usually feed on krill (*Euphausia superba*), fish and squid [28], and monitoring their diet can also provide insights into the bioaccumulation of contaminants, which is imperative for understanding environmental health worldwide.

Therefore, this study aimed to evaluate the presence of perfluorinated compounds (PFOS and PFOA), phthalates (DEHP and MEHP) and BPA in *Pygoscelis antarctica* and *Euphausia superba* from Deception Island (South Shetland Islands, Antarctica) in order to provide additional data on the occurrence of emerging pollutants in Antarctica.

## 2. Materials and Methods

**Figure 1:**
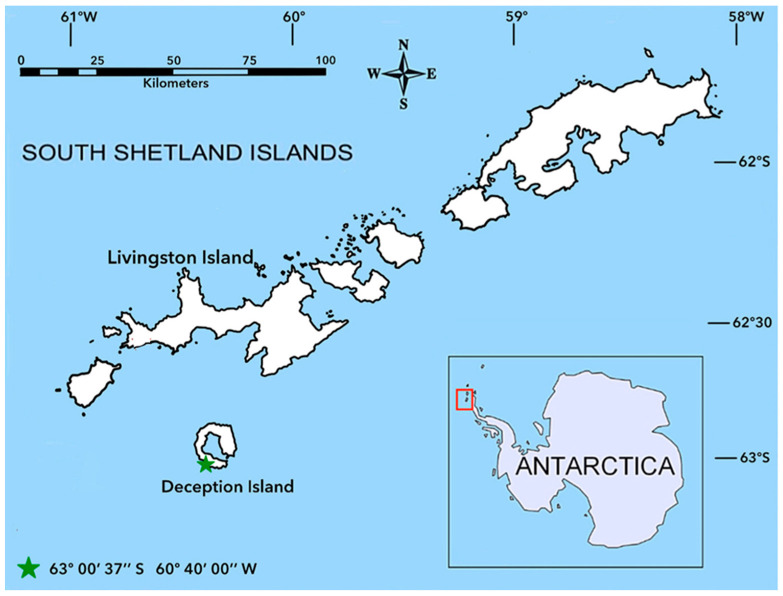
Map showing the location of the two penguin breeding colonies in the South Shetland Islands.

The concentration and tissue distribution of emerging pollutants [perfluorinated compounds (PFOS and PFOA), phthalates (DEHP and MEHP) and bisphenol A] were determined in chinstrap penguins (*Pygoscelis antarctica*) and krill (*Euphausia superba*) collected during the austral summer season of Antarctic campaigns from 2007 to 2010 from the population located on Deception Island (South Shetland Islands, Antarctica, 63°00′ S 60°40′ W) (Figure 1). At the present time, Deception Island is home to two scientific research stations, which are open only for the summer months. These are the Spanish Gabriel de Castilla and the Argentinian Deception stations. In addition to these, there are ruins of other stations that were destroyed in the last volcanic eruption in 1967, and the island’s formation is the result of a volcano that erupted and created a horseshoe-shaped crater. The flooded caldera of this volcano has given rise to a natural harbor that is unique to the area [29]. Since the beginning of the last century, human activities have been hosted in this natural refuge, mainly in the form of whale and seal hunting and the whaling industry until 1967 [30]. Nowadays, it also supports heavy traffic of ships and cruise vessels. The sampling location was Vapour Colony, situated at 63°00′ S 60°40′ W, and home to an estimated 20,000 pairs of penguins [31]. This population was chosen for the study of the previously mentioned pollutants because, based on an extensive literature review, no prior studies have been conducted on this subject at this location. Factors such as its position in the food chain, longevity, diet and other characteristics contribute to its ability to bioaccumulate and biomagnify organic contaminants. Additionally, a considerable number of samples are available for analysis. It was also considered relevant to compare with the levels most widely described in Pygosceli penguins from King George Island [32,33,34,35,36,37].

During the Antarctic campaigns from 2007 to 2010, researchers collected the carcasses of adults and chicks and preserved them frozen at −20 °C in individual polyethylene bags until they analyzed them the same year. A total of thirty-four penguin samples were analyzed: four adult and six chick specimens were studied, with samples taken from the liver, kidneys, muscles, heart and brain. Krill samples, the primary prey of penguins, were analyzed and collected from the stomach contents of penguin specimens using the stomach lavage method [38,39] (Table 1). On account of the limited quantity of chick specimens, a number of tissues were homogenized to generate 5 g pools for the assessments (Table 1).

The selected samples were subjected to a series of extraction processes, followed by the analytical determination of di(2-ethylhexyl) phthalate (DEHP), mono(2-ethylhexyl) phthalate (MEHP), perfluorooctane sulfonate (PFOS), perfluorooctanoic acid (PFOA) and bisphenol A (BPA).

The sample preparation method for perfluorinated compounds is based on that previously described elsewhere [40]. Each sample (1 g) was homogenized with Milli-Q water. One ml of the homogenized sample was taken, and 1 mL of 0.50 M tetrabutylammonium (TBA) hydrogen sulphate solution and 2 mL of 0.25 M sodium carbonate buffer were added. A liquid–liquid extraction (LLE) was conducted, adding 5 mL of methyl tert-butyl ether (MTBE) to the mixture. After centrifugation, the organic layer was transferred to a clean tube and the process was repeated. The solvent was evaporated under a nitrogen atmosphere until an aliquot was resuspended in an exact volume of methanol. Finally, the extract was passed through a nylon filter (0.20 μmm) and transferred to a chromatography vial. The samples were analyzed using high-performance liquid chromatography coupled to an electrospray ionization mass spectrometer (LC-ESI-MS). The chromatography system used was a Finnigan Surveyor Plus HPLC system (Thermo Electron Corporation, San Jose, CA, USA), consisting of a quaternary pump, a vacuum pump and an autosampler. Chromatographic separation was conducted using a Betasil C18 column (50 × 2.10 mmI.D., 5 μm). The linear ion trap mass spectrometer (Finnigan LTQ, Thermo Electron Corporation, San Jose, CA, USA) was operated in the negative electrospray mode. The limits of detection (LOD) were determined as three times the signal-to-noise ratio (S/N) and were 0.50 ng·g^−1^ for both compounds.

The method for the extraction of phthalates is based on one previously described by Takatori et al. [41]. DEHP and MEHP were analyzed using LC-ESI-MS. The equipment used was the same as that described for the analysis of perfluorinated compounds. Briefly, 1 g of each sample was taken, and 4 mL of acetone was added. The sample was sonicated, shaken and centrifuged. The organic phase was transferred to a polypropylene tube. A second extraction of the aqueous phase was conducted with 1 mL of acetone. The upper layer was retrieved and combined with the outcome of the primary extraction. Both supernatants were evaporated and resuspended in Milli-Q water (0.5 mL) and acetic acid (4 mL). The process described above was repeated 4 times with hexane. The sample was mixed and passed through 1 nylon filter (0.20 μm). The final volume was adjusted to 0.50 mL for analysis using LC-ESI-MS. A reversed-phase column (Wakosil 3C18, 2.00 × 100 mm, 3 μm) was used. MEHP was detected in the negative mode and DEHP in the positive mode. The LOD were determined as the mean value of the compounds in the blanks + 3SD and were 2.00 ng·g^−1^ for MEHP and 10.00 ng·g^−1^ for DEHP.

The method for BPA extraction is based on that previously described by Prins et al. [42]. For the analysis of total BPA, including free and inactive forms (BPA-glucuronides), 0.50 g of the sample was taken, to which 5 ng of deuterated BPA was added. The sample was digested with 1 mL of glucuronidase (2 µL·ml^−1^) at 37 °C for 12 h. It was then homogenized, and 3 mL of ethyl ether was added. The sample was shaken and centrifuged. The supernatant was transferred to a polypropylene tube and the process was repeated three times. The solvent was evaporated under a nitrogen atmosphere until an aliquot was resuspended in 0.50 mL of methanol. Finally, the extract was filtered using a nylon filter (0.20 μm). The analytical determination was performed as described by Coughlin et al. [43]. The LC-ESI-MS method in negative mode was the same as that described for the analysis of perfluorinated compounds. The analyte separation was conducted using a C18 Betasil column (50 × 2.10 mm I.D., 5 μm). The LOD was taken as the mean value of the compound in the blanks + 3SD and was 0.50 ng·g^−1^.

The analytical processes were accompanied by strict quality control. Thus, the reliability and reproducibility of the results was guaranteed by the following procedures:

- Introduction of samples in duplicate at random;
- Introduction of reagent blanks at the beginning and every 5 samples;
- Initial and periodic analysis of calibration standards. Standard solutions of the compounds were prepared at 4 different concentrations. When the upper limit of a calibration line was insufficient because levels higher than this were detected in the problem samples, higher concentration standards were prepared or the sample in question was diluted;
- Addition of internal standards for the analysis of BPA;
- Analysis of matrices spiked with known quantities of the compounds studied. Spiked matrices were used for each type of sample analyzed. Recovery percentages were 87–92% for PFOS, 85–91% for PFOA and 85–90% for phthalates and BPA.

All reagents used for sample processing and analysis were ultra-pure, and double-distilled and deionized water was obtained using a Milli-Q Plus Millipore water purifier.

The statistical treatment applied to the results obtained in this chapter was as follows: the Student’s *t*-test and its non-parametric analogue (Mann–Whitney U) were used to identify statistically significant differences in the accumulation of contaminants between adults and chicks and between predator and prey; the one-way ANOVA test and its non-parametric analogue (Kruskal–Wallis), with their corresponding post-hoc tests (Bonferroni and “least significant difference between mean ranks”, respectively), were used to identify statistically significant differences in the accumulation of contaminants in the different internal tissues of penguins analyzed. With regard to the analysis of data, a value of half the detection limit (DL/2) was assigned to samples that did not demonstrate detectable levels of pollutants. The results are presented as the mean ± standard deviation (SD) and the range from minimum to maximum on both a wet weight (w.w.) and lipid basis.

## 3. Results and Discussion

### 3.1. Relative Contributions of Different Contaminant Families in Pygoscelis Antarctica and Krill

Our findings revealed that phthalates constituted 80% and 90% of the pollutants examined in the livers of adult *Pygoscelis antarctica* and krill, respectively. Conversely, perfluorinated compounds were predominant in the muscle tissue of adults and in the liver and muscle tissue of chicks, and BPA was not found [44].

### 3.2. Perfluorinated Compounds, Phthalates and Bisphenol a Levels in Pygoscelis Antarctica and Krill

Table 2 shows the levels of PFOA, PFOS, MEHP, DEHP and BPA found in our samples. Almost all the samples (91.43%) contained PFOA, whereas PFOS was only detected in two of the samples (5.71%). MEHP was found in 25.71% of our *Pygoscelis antarctica* samples but was not found in krill. Conversely, DEHP was present at detectable levels in krill but not in *Pygoscelis antarctica* tissues. Finally, BPA was not detected in this study [44].

Compared to other pollutants, such as PCBs or metals, these pollutants have been studied far less in Antarctic organisms. Therefore, it is difficult to establish temporal trends or identify geographical differences.

In reference to PFCs, our findings on PFOS concentrations were consistent with those observed in the blood of polar skuas from Terra Nova Bay (Antarctica) in 2001 [10], which ranged from undetectable levels to 1.4 ng·g^−1^ w.w. A study of albatross livers from the Southern Ocean revealed the presence of PFOA, with amounts ranging from undetectable to 2.45 ng·g^−1^ w.w. This is analogous to or marginally lower than the levels documented in our study. However, PFOS emerged as the predominant perfluorinated contaminant in albatross livers. Concentrations were less than 5 ng·g^−1^ w.w., with a mean of 2.2 ng·g^−1^ w.w., which is higher than the majority of values recorded in our study [45]. In this same study, PFOA and PFOS were not detected in Adélia penguin blood from Antarctica. The levels of PFOS found in our study were comparable to the amounts detected in the eggs of gentoo and Adélie penguins from King George Island in 2009 [46], 0.3 and 0.4 ng·g^−1^ w.w., respectively. Nonetheless, Schiavone, Corsolini, Kannan, Tao, Trivelpiece, Torres and Focardi [46] detected quantifiable levels of PFOS in all of their specimens, whereas we solely identified this substance in 5.71% of ours. In contrast, this manuscript also reported PFOA levels (<0.2 ng·g^−1^ w.w.) that were one order of magnitude lower than those found in the tissues of our chinstrap penguins. An increase in the environmental concentrations of PFOA in the South Shetland Islands could be suggested by these findings, which could have been caused by the different collection periods. These results may also be associated with intraspecific and geographical variations, which should be examined in future research.

Arctic organisms have received more attention regarding exposure to these contaminants. Some studies have found different patterns over time for PFOS and PFOA in the Arctic. There appears to be a decline in PFOS, whereas PFOA and its precursors are either remaining constant or increasing [47]. The trends observed in the Arctic may align with our findings in Antarctica, as PFOA was detected in most samples, whereas PFOS was mostly undetected.

A remarkable outcome emerges when a comparison is drawn between the levels of PFOA identified in the present study and those previously documented in Arctic seabirds by various researchers. The levels of PFOA in the tissues of Antarctic penguins were higher than those detected in the livers or eggs of seabirds from the Arctic (less than 1 ng·g^−1^ w.w.) [48,49,50,51,52,53]. Conversely, PFOS concentrations in the livers, eggs and plasma of Arctic seabirds (range: from undetectable to 134 ng·g^−1^ w.w.; [48,49,50,51,52,53,54,55]) and aquatic birds from more populated and industrialized regions, such as the Great Lakes, North Pacific or Mediterranean Sea (range: from undetectable to 460 ng·g^−1^ w.w.; [10]) were distinctly elevated in comparison to those detected in our samples. Even though these differences are substantial, it is important to be careful when making comparisons because different species have different feeding habits. Additionally, the varying global transport pathways of PFOS and PFOA can explain their differences in mobility. PFOA demonstrates significantly higher mobility than PFOS, which is attributed to their distinct molecular structures and adsorption mechanisms. This suggests that PFAS exposure is primarily a result of the long-range transport of these substances, rather than being solely due to local anthropogenic activities [56]. In accordance with Schiavone, Corsolini, Kannan, Tao, Trivelpiece, Torres and Focardi [46], our findings substantiate the presence of PFCs in Antarctica and their pervasive dissemination. Although the levels identified in this investigation are multiple orders of magnitude lower than those that have been shown to trigger unfavorable outcomes in animals (in the range of tens to hundreds of μg·g^−1^ w.w. [57,58]), it is imperative that the health of Antarctic organisms be closely observed in the coming years, as there is a pervasive international tendency to increase the utilization of PFCs.

Regarding phthalates, the reporting of levels of these compounds in wildlife is a feature of only a very small number of studies. Although DEHP was found by Cicciol et al. [59] and Cincinelli et al. [60] in atmospheric samples from Antarctica, as well as in marine water and snow, as far as we know, no data on DEHP or MEHP levels in Antarctic seabirds or krill are available. Our results on DEHP levels in krill were in accordance with those found in a marine aquatic food web from North America (56–16,595 ng·g^−1^ lipid basis) collected in 1999 [61], but DEHP was not found in krill collected from King George Island in 2016; however, 104.3 ± 0.5 ng·g^−1^ of another phthalate, dibutyl phthalate, was found [62]. In this context, a decrease in DEHP concentrations with increasing trophic position has been reported, which aligns with our results, as we found detectable DEHP levels in krill but not in penguin tissues [61]. It has also been reported that mollusks, crustaceans and amphibians are especially sensitive to phthalates and biological effects, such as in reproduction, impairing development and inducing genetic aberrations, which can be observed at very low environmental exposure concentrations (from ng·l^−1^ to µg·l^−1^) [63]. The DEHP concentrations we found in krill show, on the one hand, the capacity of krill to absorb and bioconcentrate this substance. Furthermore, the presence of the native compound points to continuous exposure to this plasticizer, providing evidence of its presence in Antarctic marine environments. On the other hand, these concentrations suggest that there is real potential for effects of this compound on these organisms. In subsequent studies, it is imperative to incorporate a rigorous testing procedure that encompasses the effects of long-term exposure to pertinent levels of DEHP, in conjunction with its ecotoxicity, particularly when it is a constituent of intricate environmental mixtures. Both invertebrates and vertebrates rapidly metabolize DEHP to its primary metabolite, MEHP [64], which serves as a marker for DEHP exposure [65]. Although DEHP was not found in penguin tissues, MEHP was detected in samples of liver, kidney, heart and brain. If we compare our results on MEHP levels with those found in other marine organisms from different regions worldwide, the penguin tissue levels in this study were of the same order of magnitude as bubbler samples from whales in the Mediterranean Sea (1.00–99.93 ng·g^−1^ w.w. [65]) and invertebrates and fish from an urbanized area on the west coast of North America (undetected to 6.72 ng·g^−1^ w.w. [66]. Therefore, the presence of high concentrations of phthalates in Antarctic biota has been confirmed for the first time by this finding, and it should be monitored and evaluated in further studies.

With regard to the presence of BPA, we found undetectable levels of this contaminant in our samples of Antarctic penguins, despite the fact that this contaminant has been detected in atmospheric samples from polar regions [67]. This could be because there has been a decrease in the levels of BPA found in the environment, from the Asian continent to remote places such as Antarctica [67]. On the other hand, BPA has been found in the wastewaters of some Antarctic research stations [21,22]. Nevertheless, future studies should investigate the presence of BPA in Antarctic organisms, as there is currently a lack of available data on this issue.

## 4. Conclusions

Our results highlight that the organic pollutants analyzed, except for BPA, are clearly present in chinstrap penguins and krill from Deception Island.

The levels of PFOA in Antarctic penguins were higher than those reported in seabirds from the Arctic, and a potential increasing trend should be investigated in future studies.

For the first time, as far as we know, the presence of DEPH and MEHP have been reported in Antarctic penguins and krill, which contained levels similar to those found in marine organisms from a priori more polluted regions, such as North America or the Mediterranean Sea. These compounds can cause adverse effects in organisms at low exposure levels and can affect Antarctic penguins and krill. This should also be investigated in future studies.

Finally, the only way to prevent the presence of perfluorinated compounds, phthalates and BPA in Antarctica is to limit the use of plastics globally and to limit Antarctic tourism by implementing extreme control measures for tourists and scientific personnel at the local level.

## Figures and Tables

**Table 1:** The identification number assigned to the specimens collected (# sample), tissues (A = liver, kidney, muscle, heart and brain; B = liver, kidney, muscle and heart) (n.a. = not available) and, for chick samples, the number of pools and pooled specimens. The weight (g) of pooled krill samples is also reported.

# Sample	Life Stage	Weight (kg)	Tissue
1A	adult	2.5	B
2A	adult		A
3A	adult	3.5	A
4A	adult		A
1C	chick	2.6	A
2C	chick	2.1	A
3C	chick		A
4C	chick	1.8	A
5C	chick	2.0	A
6C	chick		A
Chicks			
tissue	no. of pool	no. of specimen	
liver	3	1C + 6C, 3C + 4C, 2C + 5C	
kidney	3	1C + 2C, 3C + 4C, 5C + 6C	
muscle	5 single samples	(sample no. 4C: n.a.)	
heart	2	1C + 2C + 3C, 4C + 5C + 6C	
brain	2	1C + 2C + 3C, 4C + 5C + 6C	
Krill	5.1 g		

**Table 2:** Concentrations of perfluorinated compounds, phthalates and BPA (average ± SD, min–max in ng·g^−1^ w.w. and lipid basis *, and the percentage of detectable levels) in *Pygoscelis antarctica* tissues and *Euphausia superba* from Deception Island.

Samples	PFOA	PFOA *	(%)	PFOS	PFOS *	(%)	MEHP	MEHP *	(%)
**Liver (A)**	2.5 ± 1.3 1.1–4.3	111.6 ± 60.9 49.8–195.3	(100)	<0.5	<0.5	(0)	11.0 ± 20.0 <2.0–40.9	497.5 ± 904.4 <2.0–1854.2	(25)
**Liver (C)**	2.2 ± 1.6 <0.5–3.6	69.7 ± 49.3 <0.5–112.0	(66)	<0.5	<0.5	(0)	<2.0	<2.0	(0)
**Kidney (A)**	3.3 ± 1.4 1.8–5.2	102.8 ± 45.1 57.0–165.0	(100)	09 ± 0.7 <0.5–1.8	26.5 ± 21.0 <0.5–58.0	(25)	4.8 ± 7.7 <2.0–16.4	155.2 ± 246.6 <2.0–525.1	(25)
**Kidney (C)**	3.4 ± 1.0 2.5–4.5	81.4 ± 24.5 60.3–108.3	(100)	<0.5	<0.5	(0)	50.6 ± 29.1 18.8–75.8	1217.5 ± 699.6 451.2–1822.1	(100)
**Muscle (A)**	3.4 ± 1.7 2.1–6.0	249.3 ± 125.8 150.3–432.7	(100)	<0.5	<0.5	(0)	<2.0	<2.0	(0)
**Muscle (C)**	2.3 ± 1.4 <0.5–4.3	126.6 ± 78.4 <0.5–234.4	(80)	1.7 ± 2.7 <0.5–6.5	97.3 ± 145.5 <0.5–352.5	(20)	<2.0	<2.0	(0)
**Heart (A)**	2.2 ± 1.5 <0.5–4.1	144.5 ± 101.6 <0.5–280.0	(75)	<0.5	<0.5	(0)	<2.0	<2.0	(0)
**Heart (C)**	2.8 ± 0.4 2.5–3.1	113.1 ± 16.7 101.3–124.9	(100)	<0.5	<0.5	(0)	45.3 ± 24.2 28.3–62.4	1809.3 ± 962.3 1128.8–2489.8	(100)
**Brain (A)**	1.8 ± 0.5 1.2–2.3	42.8 ± 12.5 29.2–53.7	(100)	<0.5	<0.5	(0)	25.9 ± 23.4 <2.0–47.5	609.4 ± 550.9 <2.0–1116.8	(66)
**Brain (C)**	1.4 ± 0.9 0.8–2.0	67.4 ± 43.2 36.9–98.0	(100)	<0.5	<0.5	(0)	<2.0	<2.0	(0)
**Krill**	1.3	32.8	(100)	<0.5	<0.5	(0)	<2.0	<2.0	(0)
**Samples**	**DEHP**	DEHP *	**(%)**	**BPA**	BPA *	**(%)**			
**Liver (A)**	<10.0	<10.0	(0)	<0.5	<0.5	(0)			
**Liver (C)**	<10.0	<10.0	(0)	<0.5	<0.5	(0)			
**Kidney (A)**	<10.0	<10.0	(0)	<0.500	<0.5	(0)			
**Kidney (C)**	<10.0	<10.0	(0)	<0.5	<0.5	(0)			
**Muscle (A)**	<10.0	<10.0	(0)	<0.5	<0.5	(0)			
**Muscle (C)**	<10.0	<10.0	(0)	<0.5	<0.5	(0)			
**Heart (A)**	<10.0	<10.0	(0)	<0.5	<0.5	(0)			
**Heart (C)**	<10.0	<10.0	(0)	<0.5	<0.5	(0)			
**Brain (A)**	<10.0	<10.0	(0)	<0.5	<0.5	(0)			
**Brain (C)**	<10.0	<10.0	(0)	<0.5	<0.5	(0)			
**Krill**	28.8	700.6	(100)	<0.5	<0.5	(0)			

## Data Availability

The data presented in this study are available on request from the corresponding author. The data are not publicly available due to privacy restrictions.
